# Adaptive Introgression Facilitates Adaptation to High Latitudes in European Aspen (*Populus tremula* L.)

**DOI:** 10.1093/molbev/msab229

**Published:** 2021-07-30

**Authors:** Martha Rendón-Anaya, Jonathan Wilson, Sæmundur Sveinsson, Aleksey Fedorkov, Joan Cottrell, Mark E S Bailey, Dainis Ruņǵis, Christian Lexer, Stefan Jansson, Kathryn M Robinson, Nathaniel R Street, Pär K Ingvarsson

**Affiliations:** 1 Linnean Centre for Plant Biology, Department of Plant Biology, Uppsala BioCenter, Swedish University of Agricultural Science, Uppsala, Sweden; 2 Matis Ltd, Reykjavik, Iceland; 3 Institute of Biology, Komi Science Center, Russian Academy of Sciences, Syktyvkar, Russia; 4 Forest Research, Northern Research Station, Roslin, United Kingdom; 5 School of Life Sciences, College of Medical, Veterinary and Life Sciences, University of Glasgow, Glasgow, United Kingdom; 6 Genetic Resource Centre, Latvian State Forest Research Institute “Silava”, Salaspils, Latvia; 7 Department of Botany and Biodiversity Research, University of Vienna, Vienna, Austria; 8 Umeå Plant Science Centre, Department of Plant Physiology, Umeå University, Umeå, Sweden

**Keywords:** adaptation, introgression, postglacial colonization, selective sweep, balancing selection

## Abstract

Understanding local adaptation has become a key research area given the ongoing climate challenge and the concomitant requirement to conserve genetic resources. Perennial plants, such as forest trees, are good models to study local adaptation given their wide geographic distribution, largely outcrossing mating systems, and demographic histories. We evaluated signatures of local adaptation in European aspen (*Populus tremula*) across Europe by means of whole-genome resequencing of a collection of 411 individual trees. We dissected admixture patterns between aspen lineages and observed a strong genomic mosaicism in Scandinavian trees, evidencing different colonization trajectories into the peninsula from Russia, Central and Western Europe. As a consequence of the secondary contacts between populations after the last glacial maximum, we detected an adaptive introgression event in a genome region of ∼500 kb in chromosome 10, harboring a large-effect locus that has previously been shown to contribute to adaptation to the short growing seasons characteristic of Northern Scandinavia. Demographic simulations and ancestry inference suggest an Eastern origin—probably Russian—of the adaptive Nordic allele which nowadays is present in a homozygous state at the north of Scandinavia. The strength of introgression and positive selection signatures in this region is a unique feature in the genome. Furthermore, we detected signals of balancing selection, shared across regional populations, that highlight the importance of standing variation as a primary source of alleles that facilitate local adaptation. Our results, therefore, emphasize the importance of migration–selection balance underlying the genetic architecture of key adaptive quantitative traits.

## Introduction

Local adaptation, the means by which populations of a species genetically adjust to local environments, is a powerful process of evolution. It occurs because multiple environmental factors imposing different selective pressures exist and the strength of each factor varies across habitats. When a population colonizes a new habitat, certain environmental conditions will impose higher selective pressures, whereas natural selection may be relaxed for other environmental factors. The overall shift in the selection landscape leads to local adaptation and consequent fitness trade-offs (VanWallendael et al. 2019). Two fundamental genetic sources for local adaptation, particularly in temperate forest trees that have large effective population size and low nucleotide substitution rates per unit of time, are standing variation and intra-/interspecies hybridization. Hybridization occurs when reproductive isolation is not complete between species or when species lineages that are separated geographically meet after a secondary contact. Species capable of hybridization will therefore have access to a larger pool of genetic variation that provides the raw material for selection and accelerated adaptation. At the same time, standing variation can be maintained through balancing selection (BS) within populations. The signatures of BS include increased diversity around the target of selection, differentiation between populations departing from the genome-wide average and increased linkage disequilibrium, among others (Fijarczyk and Babik 2015). When selection varies geographically, it may favor locally adapted alleles in the derived lineages or subpopulations, giving rise to genomic regions with elevated *F*_ST_ and absolute divergence *D*_XY_ (Guerrero and Hahn 2017; Wang et al. 2019).

Throughout the entire Pleistocene, range expansions and contractions of different species occurred in Europe, influencing the current patterns of diversity in many taxa (Widmer and Lexer 2001). Paleoclimate model simulations and pollen and plant macrofossil records have revealed a long-term decline in tree populations, with a threshold at the time of Heinrich Stadial (HS) 2 (24 thousand years ago [ka]), when tree populations decreased dramatically and did not recover until the end of HS1 (15 ka). At this point, there were no refugia for temperate trees north of 45°N in Europe, which means that present-day populations of temperate trees in northern Europe are essentially young, in contrast to conifers and other boreal tree species (Svenning et al. 2008). This also implies that temperate trees in northern Europe derive from southern populations, where they survived in glacial refugia until temperature and moisture conditions allowed for the recolonization of higher latitudes (Tzedakis et al. 2013). The distribution of glacial refugia across the continent and the successive contact of isolated populations following range expansions often led to hybridization events that played a major role in the evolution of forest trees and other plants (Hewitt 2004; Canestrelli et al. 2016).

European aspen, *Populus tremula*, is a dioecious temperate angiosperm tree with a very extensive range across both the European and Asian continents. Its role in many ecosystems is that of a primary colonizer for forests, growing quickly and being able to cover large areas across both different latitudes and elevations (Worrel 1995). Natural variation of *P. tremula* along a north–south gradient on the Scandinavian peninsula has been widely studied. GWAS and population differentiation analyses have identified a region on chromosome 10 harboring the *PtFT*2 locus, a gene known to be involved in controlling seasonal phenology (Wang et al. 2018), as a key contributor to climate adaptation at high latitudes in Sweden. Other loci harboring genes related to senescence processes or plant growth have also been proposed to have played a role in local adaptation, based on differences in haplotype frequencies identified with the hapFLK statistic (Fariello et al. 2013) between Swedish populations originating at different latitudes (Ingvarsson and Bernhardsson 2020).

Introgression has been hypothesized to be a major driver of adaptation in the genus *Populus*, where the lack of reproductive barriers allows for prevalent interspecies hybridizations. One such example is given by the adaptive introgression of a telomeric region on chromosome 15 from *P. balsamifera* to *P. trichocarpa* that has been implicated in climate adaptation in the recipient species (Suarez-Gonzalez et al. 2016). In the case of *P. tremula*, admixture in contact zones along the Scandinavian peninsula has been documented and, even though different genetic lineages of *P. tremula* can be defined across Europe (de Carvalho et al. 2010), no cases of adaptive introgression have been described so far in this species.

Using whole-genome resequencing data from an extensive set of Eurasian samples of *P. tremula*, we describe different aspects of the postglacial colonization, adaptation, and admixture in the species across the Eurasian continent. We dissect the population structure of the species, revealing a complex and intricate genomic mosaicism supporting and extending earlier results from much more limited data sets (de Carvalho et al. 2010). Furthermore, we studied different types of selection that underlie local adaptation of aspen. Interestingly, we observed a clear and unique case of adaptive introgression, in which the strong selective sweep previously observed on chromosome 10 that harbors a gene controlling bud set in Northern Scandinavia resulted from a recent introgression event from the Russian gene pool. Overall, our results highlight the importance both of standing genetic variation and of genomic introgression as sources of new alleles for local adaptation.

## Results

### Population Structure and Admixture

**Fig. 1.**
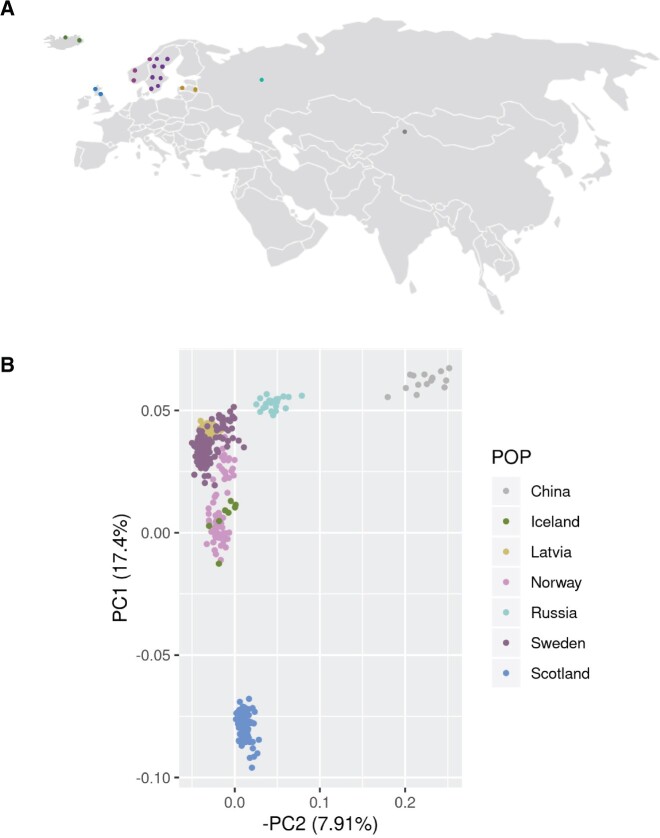
*Populus tremula* collection. (*A*) Sampling sites across the Eurasian continent. (*B*) Principal component analysis of *P. tremula* individuals, using 283,505 pruned sites across the 19 chromosomes. The principal component analysis shows evidence for at least three independent clusters of individuals, one corresponding to samples of Chinese origin, another comprising all samples collected across continental Europe and a third comprising samples collected on the British Isles (primarily Scotland).

In order to elucidate demographic and adaptive events that have accompanied the postglacial radiation of European aspen, we resequenced a large collection of individuals, covering a wide geographic range across the Eurasian continent. Our collection comprises 411 *P. tremula* individuals sampled from China, Russia, Latvia, Norway, Sweden, Iceland, and Scotland (fig. 1*A*; supplementary table 1, Supplementary Material online). Sequencing reads for all individuals were mapped against the reference *P. tremula* v2.0 genome (Schiffthaler et al. 2019) and obtained ∼20.8e^6^ single-nucleotide polymorphisms (SNPs) after several filtering steps (depth of coverage, missingness, level of heterozygosity, and batch effect caused by the incorporation of samples in the Swedish collection from two different sequencing platforms; supplementary methods and supplementary figs. 1 and 2, Supplementary Material online). It should be highlighted that the removal of false heterozygous positions did not alter the results of the downstream analyses (see Alleles Favored by Positive Selection section). Finally, we removed 48 samples that were possible hybrids or that were highly related samples, yielding a total of 363 individuals for all downstream analyses.

**Fig. 2.**
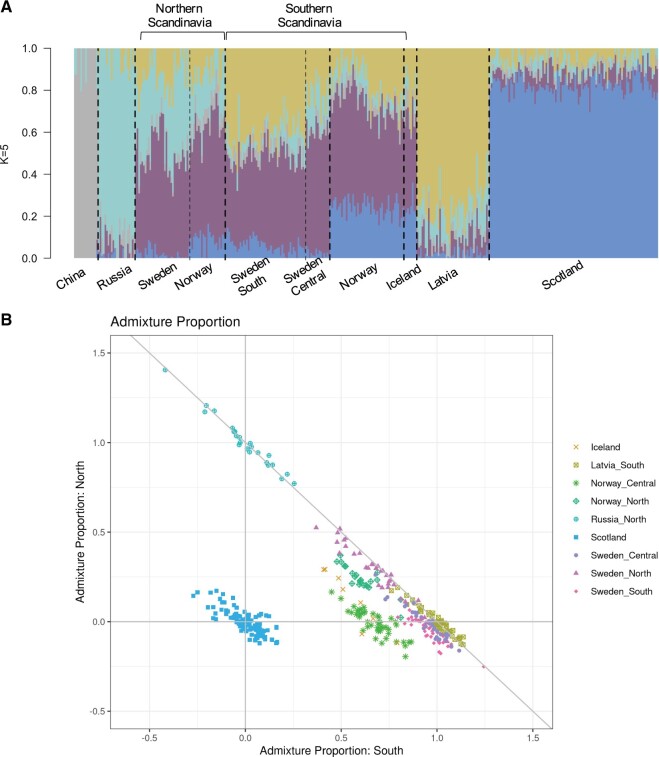
Population structure and admixture proportion. (*A*) NGSadmix plot (*K* = 5) showing the hybrid genetic background of the European populations. (*B*) Admixture plot (EIGMIX) where the Russian, Scottish and Latvian populations where selected as proxies of the ancestral postglacial gene-pools in Europe and thus, are located at the vertices of the plot. The remaining samples were positioned according to their level of admixture along the sides of the triangle.

Due to the broad geographic coverage of *P. tremula*, we first focused on estimating the population structure of the species. As depicted in figure 1*B*, a principal component analysis using 2.8e^5^ pruned, unlinked SNPs clearly separated at least three independent clusters of individuals, one corresponding to samples of Chinese origin, another comprising all samples collected across continental European, and a third comprising samples collected on the British Isles (primarily Scotland). A deeper analysis of the composition of the species structure with NGSAdmix revealed five different ancestral populations and different levels of hybridization in particular geographic regions. Chinese and Russian samples, represented by solid colors in figure 2*A* (gray and aquamarine, respectively), did not show strong signs of admixture with European samples. However, when moving toward Western Europe, admixture events occur and we can recognize three additional populations: a Scandinavian (purple), a Central-European (Latvia, yellow), and a Western European (Scottish, blue) population. Interestingly, the genomic background of the Scandinavian samples (from Norway and Sweden) is a complex mosaic of up to four populations, confirming earlier results based on microsatellite data (de Carvalho et al. 2010) and providing additional evidence for several waves of migration that led to the colonization of the Scandinavian peninsula.

Given these observations, we took a closer look at the genomic mosaicism of the Scandinavian population. We used EIGMIX, taking Russian, Scottish and Latvian populations as proxies of the ancestral Siberian, Western and Central European populations, respectively (fig. 2*B*, see also de Carvalho et al. [2010]). These populations correspond to the vertices of the graph in figure 2*B*, whereas the remaining samples were positioned according to their level of admixture along the sides of the triangle. This analysis, consistent with the observations derived from the NGSadmix analysis, shows that individuals collected in Northern Scandinavia have on average 26% of Russian origin with a maximum value of 52% in Sweden (SWASP_108 and SWASP_115) and 37% in Norway (NO_MIR20). Although the second highest ancestral component ($\bar{x}$ = 0.66) in Northern Scandinavia corresponds to the Central European population, in the samples from northern Norway there is up to 20% of ancestry from the Western European population. The opposite trend was observed in Southern Scandinavia, where the dominant ancestral component is Central European ($\bar{x}$0.75), with less than 10% of Russian admixture and up to 18% and 44% of Western European ancestry in samples from Sweden and Norway, respectively. This Western European genomic component (colored in blue in fig. 2*A*), which is strongly represented among the Scottish and Norwegian samples, derives from an additional ancestral population, probably located between the Iberian and Italian peninsulas that we did not cover in our sampling. These observations also fit the results from earlier analyses based on microsatellite data (de Carvalho et al. 2010). Icelandic samples largely overlap with Norwegian individuals, suggesting that the population in Iceland has been recently introduced and has a clear Norwegian origin.

These observations were also supported by *F*_ST_ estimations (table 1), as we obtained a small dispersion of values when calculating pairwise comparisons between Russia and Northern Scandinavia (mean *F*_ST_ = 0.003, maximum at 0.09), or Latvia and Southern Scandinavia (mean *F*_ST_ between Latvia and Sweden of 0.002, maximum at 0.07 and between Latvia and Norway of 0.004, maximum at 0.1), whereas comparisons between populations without signs of admixture, such as China and Russia, reached values as high as 0.35, which approach *F*_ST_ values observed between closely related aspen species.

**Table 1.** Genome-wide *F*_ST_ Comparison.

		CH	ICE	LV	NOC	NON	NOS	RU	SWC	SWN	SWS	UK
*F*_ST_	CH	—										
	ICE	0.014	—									
	LV	0.012	0.003	—								
	NOC	0.016	0.003	0.005	—							
	NON	0.013	0.002	0.004	0.003	—						
	NOS	0.016	0.003	0.004	0.003	0.003	—					
	RU	0.012	0.006	0.005	0.007	0.005	0.007	—				
	SWC	0.016	0.004	0.003	0.004	0.003	0.003	0.006	—			
	SWN	0.012	0.003	0.004	0.004	0.002	0.003	0.004	0.001	—		
	SWS	0.011	0.002	0.002	0.003	0.002	0.002	0.005	0.001	0.002	—	
	UK	0.010	0.002	0.009	0.004	0.005	0.004	0.008	0.004	0.007	0.008	—

Note.—CH, China; ICE, Iceland; LV, Latvia; NOC, Central Norway; NOCN, Northern Norway; NOS, Southern Norway; RU, Russia; SWC, Central Sweden; SWN, Northern Sweden; SWS, Southern Sweden; UK, Scotland.

### Genomic Introgression

**Fig. 3.**
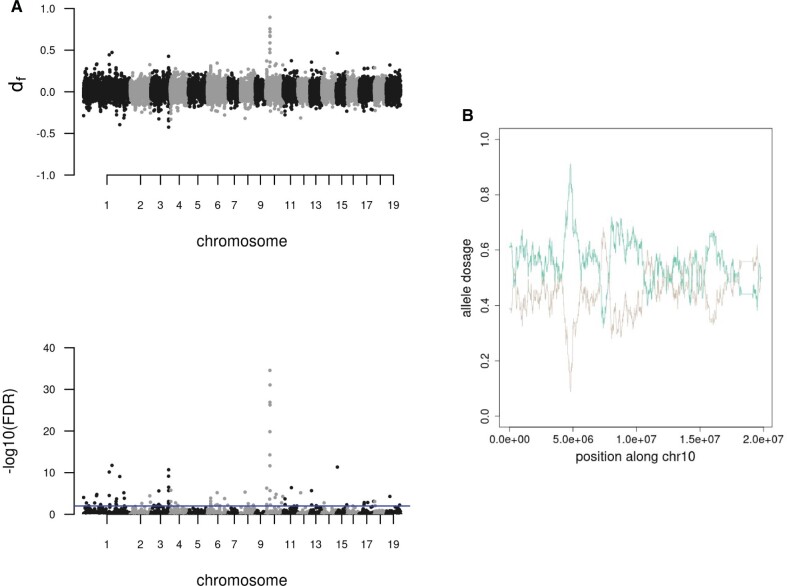
Introgression signals in Scandinavia from its ancestral gene pools. (*A*) Gene flow from Russia using a four-taxon tree hypothesis. (*B*) Allele dosage along chromosome 10 estimated with ELAI. In green, the dosage from the ancestral Russian population; in brown from the Latvian population. We observed a unique region encompassing 4.5–4.9 Mb on chromosome 10 with a peak introgression signal [*d*_f_ = 0.89 (FDR = 1.33e^−50^)] and a predominant Russian ancestry.

We scanned the genome of *P. tremula* for potential introgression events that could have occurred given the admixed background we observed in Scandinavia. We followed a four-taxon approach for these scans, keeping the Chinese population as an outgroup in all our comparisons. We calculated the *f*_dM_ introgression statistic (Malinsky et al. 2015), and the basic distance fraction, *d*_f_ (PopGenome; v2.7.1), which combines both *f*_d_ and distance estimations and that is less sensitive to the timing of gene flow. Both estimators give positive values when introgression occurs between P3 (donor population: Russia or Latvia) and P2 (Northern Scandinavia), and negative values if it occurred between P3 and P1 (Southern Scandinavia), on a scale from −1 to 1. When evaluating gene flow from Russia into Scandinavia, we obtained *f*_dM_ and *d*_f_ mean genomic values of −0.008 and 0.014, respectively (fig. 3*A*). After false discovery rate (FDR) correction at a threshold of 0.01, one region on chromosome 10, spanning from 4.5 to 4.9 Mb had a highly significant introgression signal, reaching values of *f*_dM_ = 0.85 (FDR = 3.6e^−3^) and *d*_f_ = 0.89 (FDR = 1.33e^−50^). As expected, there were differences in *D*_XY_ and *F*_ST_ patterns between subpopulations at this region: *F*_ST_ is remarkably high between Nordic subpopulations and any other geographic location, whereas *D*_XY_ decreases when northern Norwegian, Swedish, and Russian subpopulations are contrasted (supplementary fig. 3, Supplementary Material online). Introgression on chromosome 10 was further validated applying efficient local ancestry inference (ELAI) on each individual from the north of Sweden. This method implements a two-layer HMM (hidden Markov model) to infer local ancestry of admixed individuals without prior definition of window sizes or haplotype phasing, returning the most likely proportions of ancestry at each variable position of the chromosome. Running ELAI on the three hybrid populations we observed that at the genome-wide level, the allele dosage difference (ADD) is 0.14 (q.75 = 0.21, max = 0.85) in Northern Sweden, 0.11 (q.75 = 16, max = 0.52) in Southern Sweden, and 0.25 (q.75 = 0.36, max = 0.77) in Southern Norway (supplementary fig. 4, Supplementary Material online). Furthermore, ELAI confirmed that, although chromosome 10 shares alleles from both ancestral or training populations and on average, northern individuals have 44% and 56% admixture proportions from Latvian and Russian populations, respectively, the region encompassing 4.5–4.9 Mb has a predominant Russian ancestry where the highest ADD is observed (fig. 3*B*; supplementary fig. 5, Supplementary Material online).

In terms of gene content, two relevant loci annotated as *Heading-date 3A*-like, or *Flowering Locus T* (*PtFT2*) genes, are encoded in this introgressed genomic block: *Potra2n10c20839* (4,772,117–4,776,457 bp) and *Potra2n10c20842* (4,789,807–4,792,846 bp) and the region surrounding these two genes is known to have been subject to a selective sweep in the northern Swedish population (Wang et al. 2018).

### Alleles Favored by Positive Selection

**Fig. 4.**
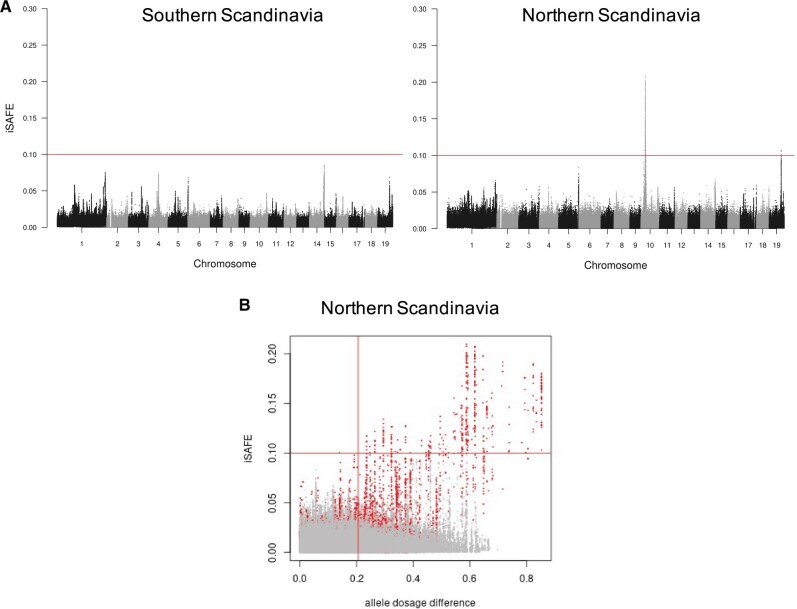
Positive selection signals in Nordic populations reveal a selective sweep on chromosome 10. (*A*) Genome-wide scan using iSAFE to identify variants favored by evolution in individuals from the southern part of Scandinavia (left panel) and northern part of Scandinavia (right panel). The red line shows the theoretical threshold of 0.1 to consider significant signals of selection. (*B*) Plot of the iSAFE signal and ADD across the 19 chromosomes in the Northern Scandinavian population. SNPs corresponding to chromosome 10 are highlighted in red; lines represent the iSAFE threshold of significance (*h* = 0.1) and a cutoff = q0.75 in ADD (*v* = 0.21).

A good way to understand local adaptation without necessarily having phenotypic data is to look for signs of positive selection in different populations across a species range. Previous evidence of selective sweeps has shown a particularly strong signal on chromosome 10, in a region encompassing the two *Flowering Locus T* homologs (Wang et al. 2018). This region is strongly implicated in the capacity of Nordic populations to adapt their seasonal phenology to the more variable day lengths and shorter growth seasons experienced at norther latitudes. We scanned the genome of *P. tremula* using a novel method to detect mutations favored by selection, iSAFE (integrated selection of alleles favored by evolution; Akbari et al. 2018). We calculated iSAFE scores for each European population and also contrasted Northern versus Southern populations in Scandinavia in a case/control analysis mode. The highest iSAFE signal (iSAFE = 0.22) was located on chromosome 10, in the region spanning 4.5 to 4.9 Mb where iSAFE values reached the recommended threshold of 0.1. The high iSAFE values were reached not only in Swedish individuals but also when we combined samples originating in Northern Norway, Northern Sweden, and Russia (fig. 4*A and B*, supplementary fig. 6, Supplementary Material online), implying that the sweep is shared among these populations derived from more northern latitudes in the Eurasian continent. As mentioned before, this region is centered around two loci annotated as Heading-date 3A-like, or *PtFT*2, in agreement with previous analyses that have associated *PtFT*2 with climate adaptation in *P. tremula* (Wang et al. 2018). In addition, we evaluated the relationship between iSAFE values and ADDs between ancestral population proxies and the Northern Scandinavian individuals. At the genome-wide level, we observed outlier SNPs in both iSAFE and ADD values only on chromosome 10 (fig. 4*C*, SNPs highlighted in red), confirming the uniqueness of this adaptive introgression event in the Scandinavian peninsula.

### Balancing Selection and Functional Enrichments

**Fig. 5.**
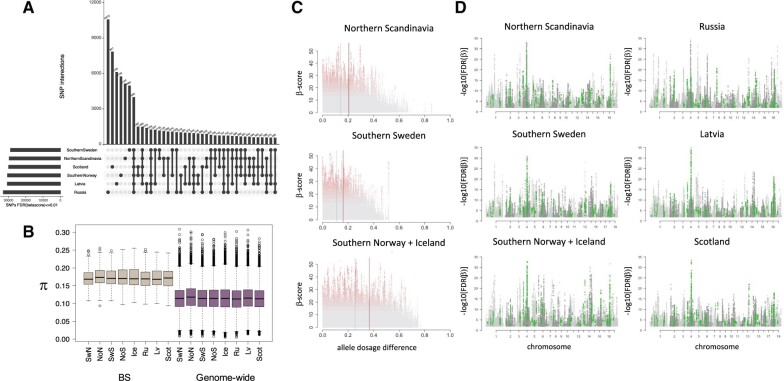
Balancing selection signal in *Populus tremula*. (*A*) We obtained the β-scores for each hybrid and the putative ancestral populations using betascan. At a significance threshold of FDR < 0.01 in each population, we calculated the intersection of SNPs and identified 4K sites shared by all the six groups. (*B*) Comparison of genetic diversity π between BS regions and genome-wide values. The BS regions are defined as those 10-kb chromosome bins with at least 15 BS sites belonging to the 4K set. (*C*) Relationship between β-scores and ADD in the hybrid populations. Blue lines show the average ADD whereas red lines the q0.75 cutoff. β-Scores are colored following a gray–red gradient according to their significance (FDR). (*D*) Manhattan plots showing balancing selection signals in the hybrid and ancestral populations. The plots are built with BS significant sites (FDR < 0.01) from each population; in green, we highlight those 1,248 sites that are shared between all the evaluated groups that do not show high ADD in the hybrid populations.

A closer look at the iSAFE patterns in the different subpopulations revealed several strong signals that 1) did not reach the accepted significance threshold to be confidently classified as regions targeted by positive selection or 2) were not geographically limited, that is, they are present in at least two distant subpopulations (supplementary fig. 8, Supplementary Material online). This suggests that another selective force may be acting to maintain alleles at high frequencies, for instance, BS. In order to evaluate this possibility, we ran betascan (Siewert and Voight 2017) on all 19 chromosomes, dividing the collection in geographic zones according to the admixture patterns observed in figure 2: Northern Scandinavia (Northern Norway and Sweden), Southern Sweden, Southwestern Scandinavia (Southern Norway and Iceland); we also ran betascan on the proxies of the ancestral populations, Russia, Latvia and Scotland. We identified 4,000 SNPs with significantly high β-scores (FDR < 0.01) shared by all the subpopulations in our collection (fig. 5*A*) and thus correspond to old species-specific BS sites. Of these, 743 fell within a predicted gene model and only 71 genes were identified having at least one of these significant signals. Given the high rate of intergenic BS sites that could have *cis*-effects on up/downstream genes, we binned the BS signal in 10-kb tracts along each chromosome and evaluated the gene content in each window displaying at least 15 BS sites. Under this criterion, the number of encoded gene models putatively affected by BS sites increased to 105. We ran gene ontology enrichment analyses on these protein-coding gene models and identified 14 enriched categories (classic Fisher, *P* < 0.05; supplementary table 4, Supplementary Material online) such as protein modification by small protein conjugation, gene silencing, macromolecule metabolic process, and vesicle mediated transport.

The effect of BS on these 10-kb regions was also evidenced by comparing diversity patterns (π), with an average value of 0.17 on the BS bins versus 0.11 at the genome-wide level (fig. 5*B*); average *F*_ST_ values did not differ between BS-affected regions and genome-wide estimations, however (supplementary fig. 3 and supplementary table 3, Supplementary Material online). Given the admixture patterns in the species, we contrasted the behavior of β-scores and ADD in each hybrid population to evaluate the effect that hybridization has in the movement and conservation of trans-lineage alleles. We observed that significant β-scores belong to not only SNPs with no recent patterns of admixture, that is, SNPs with negligeable ADD, but also SNPs whose origin can be traced to one of the donor populations, that is, SNPs with higher ADD (top 25% of ADD marked with a red line in fig. 5*C*). The intersection between the set of 4K common SNPs previously described with sites displaying low ADD varied between hybrid populations: 2,496 sites in Northern Scandinavia, 1,986 in Southern Sweden, and 3,138 in Southwestern Scandinavia, but only 1,248 were common to all populations (highlighted in green in fig. 5*D*, supplementary fig. 9*A*, Supplementary Material online) and captured very high BS signals (supplementary fig. 9*B*, Supplementary Material online).

We took a deeper look at gene models in close proximity to SNPs with high β-scores and high ADD in hybrid populations, as the bias in allele dosage from any of the donor population could give insights on the beneficial impact of those alleles (supplementary fig. 10, Supplementary Material online). In the Southern Swedish group, we identified an outlier region on chromosome 15 (approx. position 6.87 Mb) harboring 123 significant intergenic sites with β-score_[FDR]_ < 0.01 and ADD ≥ 0.5 (dosage form donor A_(Latvia)_ = 0.76; dosage form donor B_(Russia)_ = 0.24), where the predicted up/downstream gene models correspond to Potra2n15c28701/02, annotated as presenilin-like and exocyst subunit Exo70 family protein-coding genes, respectively. Similarly, in the Nordic populations of Scandinavia, we identified regions of Russian ancestry with SNPs displaying high β-scores on chromosome 4 (approx. position 5.4 Mb; dosage from donor A_(Latvia)_ = 0.29; dosage from donor B_(Russia)_ = 0.71) only 3 kb downstream of the gene model Potra2n4c9007 annotated as a gibberellin 3-beta-dioxygenase coding gene, and on chromosome 19 (14.244 Mb; dosage form donor A_(Latvia)_ = 0.29; dosage form donor B_(Russia)_ = 0.71), upstream of gene model Potra2n19c34341, annotated as and autophagy-related protein 13 coding gene.

### Demography and Secondary Contacts

**Fig. 6.**
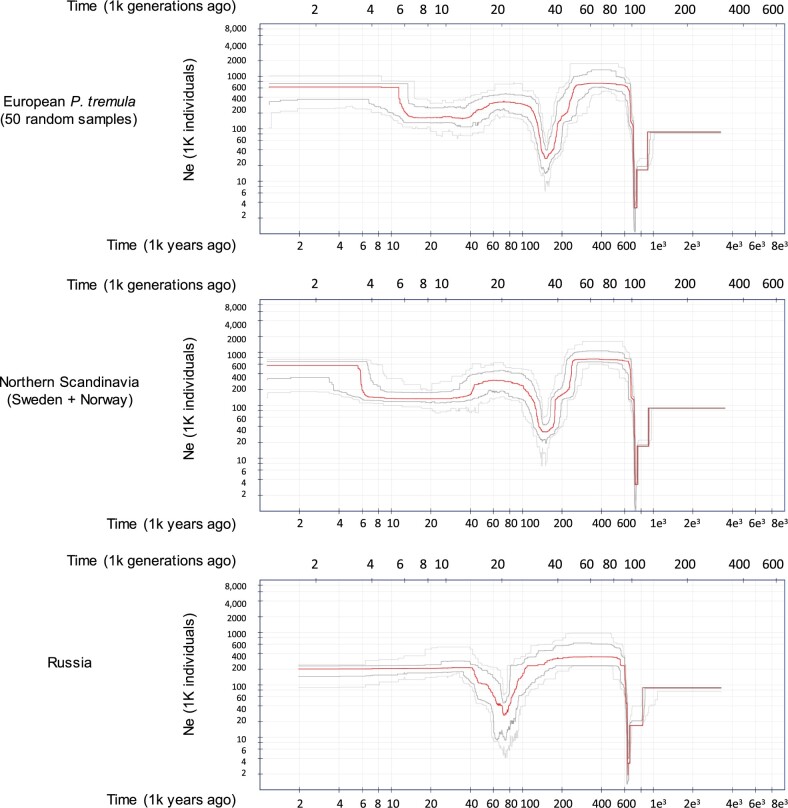
Demographic changes in *Populus tremula*. Changes in *N*_e_ across generations were simulated on three subpopulations using StairwayPlot. A sharp decrease in *N*_e_ around 700–800 ka was observed across all subpopulations, which means it was a bottleneck that affected the entire species and that predated its dispersal in the Eurasian continent. A second bottleneck occurred approximately 150–170 ka in the populations from Central Europe and Scandinavia, and a third, mild bottleneck spanned from approximately 10 to 35 ka, a period representing the last glacial era in the Northern hemisphere.

The expansion of *P. tremula* across the Eurasian continent provides a good scenario to test for changes in population sizes across time. We evaluated population size changes through time using the stairway plot method, which is based on the composite likelihood of the site frequency spectrum (SFS) of the species and/or selected subpopulations. We used 50 random samples from our collection to generate the overall behavior of the species, as well as for specific collection sites (Russia and Northern Scandinavia). We observed the strongest reduction in *N*_e_ ∼700–800 ka and this sharp decrease in *N*_e_ was observed across all subpopulations, which means it was a bottleneck that affected the entire species and that predated its dispersal in the Eurasian continent. A second bottleneck occurred ∼150–170 ka in the populations from Central Europe and Scandinavia, and a third, mild bottleneck spanned from ∼10–35 ka, a period representing the last glacial era in the Northern hemisphere. These two bottlenecks were not present in the Russian subpopulation and instead, we only observed a second decrease of *N*_e_ around 60–80 ka in this population (fig. 6).

**Fig. 7.**
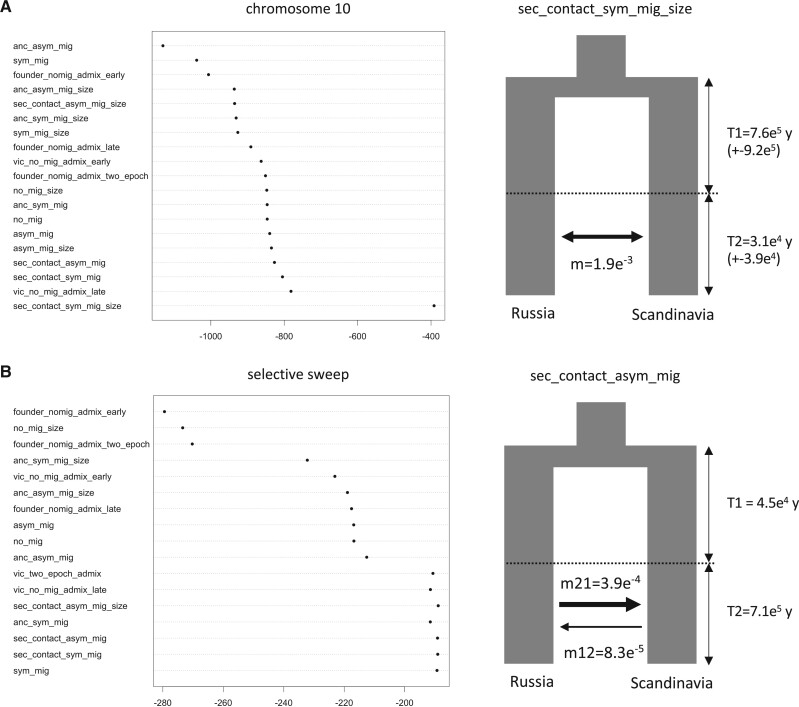
Likelihood of demographic models based on the SNP data. (*A*) Modeling using SNPs observed along chromosome 10, where the most strongly supported model included divergence in isolation with continuous symmetrical secondary contact and an instantaneous size change (sec_contact_sym_mig_size). (*B*) Modeling using SNPs comprised within the selective sweep in chromosome 10. At the sweep level several models were equally supported, with divergence in isolation with continuous asymmetrical secondary contact (with or without instantaneous size change) being the most significant models. Extrapolating the results of these simulations to time units showed that secondary contact between Russian and Scandinavian populations was dated to around 30 ka (±3.9e^4^), whereas the divergence of both lineages occurred 760 ka. Given the type of sites considered in panel *B*, the conversion of divergence times should neither be compared with the results from the entire chromosome, nor taken as actual divergence of the populations; Ne estimations are shown in supplementary table 5, Supplementary Material online.

Given the admixed nature of the Scandinavian individuals, we also evaluated 19 different demographic models including simple models of divergence with and without migration, models with instantaneous size changes, ancient migration or secondary contact, ancient migration plus instantaneous size change, and island models of vicariance and founder events. We used two levels of resolution for this analysis: We ran the 19 models at the chromosome level (supplementary figs. 11 and 12, Supplementary Material online), using unlinked sites from chromosomes 8 and 10, and also, at a region-targeted level, using the SNPs derived from the segment of chromosome 10 that we hypothesize is the result of adaptive introgression in the Northern Scandinavian population. For these analyses, we considered two populations from our collection, Scandinavia and Russia, in order to identify the most probable site of origin of the selective sweep on chromosome 10. While using all unlinked polymorphic sites on chromosomes 8 and 10 (fig. 7; supplementary fig. 13, Supplementary Material online), we consistently observed that the most strongly supported model included divergence in isolation with continuous symmetrical secondary contact and an instantaneous size change (sec_contact_sym_mig_size). Interestingly, at the sweep level, several models were equally supported, with divergence in isolation with continuous asymmetrical secondary contact (with or without instantaneous size change) being the most significant models. Not only was asymmetrical migration detected, but also it was stronger from Russia into Scandinavia than in the other direction (supplementary fig. 14, Supplementary Material online). After extrapolating the results of these simulations to time units, the secondary contact between Russian and Scandinavian populations was dated to around 30 ka (±3.9e^4^), whereas the divergence of both lineages occurred 760 ka, consistent with the first bottleneck seen at the species level in the stairway plot analyses.

## Discussion

### Aspen Populations: A Mosaic of Ancestral Gene Pools

The large number of aspen individuals included in our collection allowed us to dissect the genetic mosaicism of the species across a large geographic scale. Intraspecies admixture in *P. tremula* is not a novel idea, in fact, it has previously been documented between divergent lineages in Europe using data from microsatellite loci (de Carvalho et al. 2010). These results, first described in 2010, are, to a certain extent, corroborated by our current analyses but we have also been able to study the hybridization and introgression patterns at much finer scales across the genome of *P. tremula*. The NGSAdmix analysis identified five genetic components that explain the population structure along the East–West geographic axis in Eurasia, corresponding to Eastern Asia (China), Russia, Scandinavia (Sweden and Norway), Eastern European (Latvia) and what we suggest may correspond to Central and Western Europe (Scotland and Southern Norway + Iceland). Although Chinese and Russian individuals appear to be partly isolated from Western populations (genome-wide *F*_ST_ values between China and Western Europe ∼0.01, an order of magnitude higher than any other intraspecies pairwise comparison), the genomic mosaicism is evident particularly in Scandinavia, where the population can be divided into at least two different groups that follow the latitudinal gradient along the peninsula. Individuals from Northern Scandinavia have a strong Russian ancestral component, whereas those from Southern Scandinavia have strong influence from both Eastern and Central/Western European population. The observations of two independent routes of colonization of Scandinavia, one from the south and another one from the northeast along the ice-free Norwegian Atlantic coast, agree with findings in other organisms, such as brown bears (reviewed by Rosengren [2015]) and humans (Günther et al. 2018).

de Carvalho et al. (2010) considered Scottish and Russian subpopulations as proxies for the ancestral populations contributing to the admixed Swedish population. In our analyses, we extended this idea by using the Russian, Latvian, and Scottish populations in a triple model of hybridization that gave us a higher resolution for identifying admixture routes of the Scandinavian population. In the south of Scandinavia, we could differentiate two groups of individuals: one in Sweden with a clear Latvian component, and one in Norway not only with Latvian but also with a Scottish component. This could likely be explained by independent waves of colonization from the south of the peninsula, where Swedish and Norwegian populations were kept partially isolated by the Scandinavian Mountains, or Scandes, that run along the entire peninsula. In line with previous observations, the individuals from the Scottish populations are largely independent from individuals derived from Central Europe. Unfortunately, the lack of samples from Central Europe and the Iberian Peninsula in our collection makes it difficult to identify the origin of this population, although de Carvalho et al (2010) identified that trees from Spain constitute a different lineage themselves, possibly due to human-mediated disturbance. Finally, it is clear that the relatively few aspen clones that occur in Iceland are all recent introductions to the island of individuals with a Norwegian origin.

Many very interesting discussions have been carried out in recent years regarding paleographic migration trajectories of trees in Europe (Wetterich et al. 2011; Westergaard et al. 2019). By tracking plant macrofossil and pollen records, it has been suggested that a long-term decline in tree populations occurred around 24 ka followed by a recovery in population sizes approximately 15 ka (Tzedakis et al. 2013). The demographic changes that have affected *P. tremula* across the Eurasian continent vary along the East–West axis. The populations from Western Europe have undergone two episodes of bottleneck and subsequent recovery, one around 150 ka and another one during the Middle Pleniglacial period, from which it recovered to its current effective population size between 12–15 ka (fig. 6). On the other hand, the Russian population experienced a single severe bottleneck around 80 ka, from which it recovered before the last glacial maximum (LGM) (fig. 6). The asymmetry in the distributions of tree species along the West to East axis in Europe has been also highlighted by paleobotanical evidence and climate simulations. Western Europe generally lacked tree species north of 46N, whereas higher growing-season warmth in Eastern Europe resulted in increased permafrost thaw and higher water availability so that small populations of boreal trees were able to survive up to approximately 49.8N. The lack of a second bottleneck during the LGM in Russia suggests that the split of the European lineages of aspen predated the glacial maximum and suggests different colonization routes of Russia from southern aspen populations located in far eastern Europe and central Asia.

### Gene Flow from the East Facilitated Adaptation to Extreme Latitudes

Evidence of interspecies hybridization in poplars has been extensively documented in recent years (Suarez-Gonzalez et al. 2016; Chhatre et al. 2018) as well as intraspecies gene flow in *P. tremula* (Fussi et al. 2010). Under these circumstances, we can think of two opposite outcomes of gene flow: It can either homogenize populations and thus interfere with processes of local adaptation, or it can introduce genetic variation at higher rates than mutation would in the same time frame, providing a source of novel alleles. If any of those foreign alleles are permanently incorporated in a population as a result of gene flow and successive backcrossing, a process defined as introgression, this can increase the fitness of the recipient population and thus, can be referred to as adaptive introgression. Compared with neutral introgression, where alleles can be lost by drift, adaptively introgressed alleles are maintained by selection that can lead to fixation in the recipient population (Burgarella et al. 2019). The role of introgression for species adaptation and evolution has been recognized in animals (reviewed by Hedrick [2013]), humans (Gouy and Excoffier 2020), and plants (Bechsgaard et al. 2017; Hohmann and Koch 2017). A recent clear example of adaptive introgression was reported between two species of cypress in the mountainous region of the eastern Qinghai-Tibet Plateau, where loci from *Cupressus gigantea* introgressed into *C. duclouxiana* and thereby facilitated adaptation in *C. duclouxiana* to cooler and drier conditions at higher latitudes and elevations (Ma et al. 2019).

As several of the populations in our aspen collection are hybrids, we evaluated the occurrence of adaptive introgression events between lineages. One noteworthy genomic region was identified as it displayed clear patterns of introgression between Northern Scandinavia and Russia that were detected using different estimators of introgression (*d*_f_, *f*_dM_, and local ancestry; fig. 3) and that we could validate using patterns of genetic diversity and differentiation (supplementary fig. 6, Supplementary Material online). This genomic tract is located on chromosome 10 and comprises ∼500 kb surrounding two *FT* homologs that have already been implicated as a key component for seasonal adaptation of phenology in aspens to high latitudes in Sweden (Wang et al. 2018). Not only did the genomic tract display a clear signal of positive selection in Sweden, corroborating earlier studies (Wang et al. 2018), but it also showed strong signs of selection in other high-latitude populations, including northern Norway and Russia (iSAFE > 0.2; fig. 4).

In order to determine the population of origin of the adaptive allele in this region on chromosome 10, we used demographic modeling to test for different patterns of contact between ancestral populations and directionality of the hypothesized gene flow. First, using a set of unlinked SNPs derived from two independent aspen chromosomes (chr10 and chr8), we observed that Russian and Northern Scandinavian populations have experienced symmetric migration following a secondary contact, making it possible for backcrossing to occur and thereby promoting introgression between the two populations. Second, using information only from the adaptively introgressed region on chromosome 10, we confirmed that, indeed, the secondary contact models are the most strongly supported (together with vicariance and late admixture) and that the migration in this region was stronger from Russia into Scandinavia than in the opposite direction (supplementary figs. 13 and 14, Supplementary Material online). These analyses strongly suggest that regions surrounding the two FT homologs have been adaptively introgressed from the Russian population into Northern Scandinavia in the recent past, thereby facilitating adaptation in *P. tremula* to grow under high latitude conditions.

At the phenotypic level, there is convincing evidence that the *FT*2 allele present in Northern Scandinavia displays partial dominance (Wang et al. 2018). Trees in the northern part of Scandinavia carrying the northern *FT*2 allele initiate growth cessation about 30 days earlier than trees carrying the southern allele. Trees carrying the northern allele cannot grow in Southern Scandinavia as their critical photoperiod for growth is never reached (Michelson et al. 2018). This implies that the Nordic allele could have emerged and been kept in the Russian population in a heterozygous form during the last glaciation. The degree of dominance influences the evolutionary dynamics of alleles in diploid populations as the fixation probability of a newly arise (partially) dominant beneficial allele is higher than for a recessive allele in static or slowly changing environments, a process known as Haldane’s sieve (Devi and Jain 2020). Furthermore, recent modeling suggests that the relationship between dominance and selection coefficients arose as a natural outcome of the functional importance of genes (i.e., degree of connectivity in protein networks) and their optimal expression levels (Huber et al. 2018). Given the central role of *FT*2 in regulating the plant phenology signaling pathways and the fine tuning of its expression along plant development, we can speculate that the effect on fitness of the Nordic allele allowed a rapid increase in frequency after the colonization of the Scandinavian peninsula, where it was strongly selected for, allowing a rapid local adaptation to high latitudes. It is worth noting that despite the high recombination rate in *P. tremula* (Apuli et al. 2020), the introgression signal has not been broken and is detectable in a long tract of ∼500 kb, reinforcing the idea of recent contacts and hybridizations between Nordic populations.

### Other Paths to Local Adaptation

A major genetic process contributing to local adaptation in many populations is that of selective sweeps. Selective sweeps result in the loss of genetic variation in the neighboring regions of a newly adaptive allele or mutation as selection drives this allele to fixation. Selective sweeps generally fall into two categories, hard sweeps and soft sweeps, depending on the origin of the adaptive allele. Hard sweeps are the result of positive selection acting upon a newly arisen beneficial mutation before divergence of lineages, thus the same variation in neighboring haplotypes is driven to high frequencies alongside the adaptive allele. Conversely, a soft sweep occurs from standing variation so that haplotypes surrounding a future beneficial allele have had time to diverge before the onset of positive selection, meaning that the haplotypes that hitchhike together with the beneficial allele may differ between lineages or populations (Messer and Petrov 2013; Hermisson et al. 2017).

Our scans for positive selection using the iSAFE algorithm revealed a very strong signal of a selective sweep located on chromosome 10. Although we also observed other regions across the aspen genome that seemly have experienced selective sweeps, the calculated iSAFE values in these regions were generally too weak to pass the significance threshold and did not display a clear geographic pattern, which precluded them from being interpreted as selective sweeps involved in conferring local adaptation. A natural conclusion from the lack of nonneutral outliers in our analyses is that local adaptation in aspens is primarily polygenic and/or driven by natural selection acting on standing variation rather than from new mutations that would induce hard sweeps, as is the case for the hard sweep identified on chromosome 10.

The role of standing variation in adaptive evolution has been widely debated. As highlighted by de Carvalho et al. (2010), beneficial alleles that are present as standing variation are generally older than new mutations, implying they could have undergone a selective filter during past environmental conditions or in different parts of a species range. These polymorphisms can be maintained for a long time due to BS that persists for many generations and minimizes the effect of drift. When we think of temperate tree species, such as aspens, that have experienced several population contraction and expansion cycles throughout the last millennia, such “preselected” standing variation could represent a very useful pool for adaptive alleles that can rapidly be brought together to mediate local adaptation to novel environmental conditions encountered during range expansion. For instance, one climatic consequence for plants during the LGM was water stress due to low CO_2_ concentrations and the presence of permafrost. Even if boreal trees can grow on continuous permafrost, other factors such as soil texture or timing of the spring–summer thaw determine the amount of water available for growth, thus influencing the species distribution (Tzedakis et al. 2013).

We evaluated the importance of standing variation for local adaptation in aspen by scanning the genome for signals of BS. The combination of significant β-scores, low ADD, and the increased genetic diversity (π) in regions across multiple chromosomes shared by all the aspen lineages, indicated that BS has maintained ancestral polymorphisms in the species. Indeed, excess of diversity around selected loci may be due to the retention of ancestral polymorphisms or due to the accumulation of derived polymorphisms, which happens faster than expected under neutrality. The former is expected if the same allelic lineages have been evolving under BS for a long time, with drift and mutation determining polymorphism within each lineage. The latter would occur if new, more recent instances of BS appear, facilitating the establishment of new neutral polymorphisms (Fijarczyk and Babik 2015); given that our sampling belongs to one single species across the Eurasian continent, we can speculate it is the second scenario which has produced the excess of diversity. As reported in other species, the BS regions identified in aspens harbor potentially interesting genes for mediating local adaptation. Of particular relevance was the observation of an enrichment of genes in these genomic regions related to vesicle mediated transport and protein modification, as they are both important mechanisms in plants to cope with abiotic stress (Kosová et al. 2018; Wang et al. 2020).

Furthermore, the detection of strong BS signal among SNPs found in genomic regions with clear admixture patterns (supplementary fig. 12, Supplementary Material online), indicates that hybridization events have also allowed the spread of adaptive alleles across the Eurasian continent. For instance, gene models annotated as autophagy-related protein 13 coding gene on chromosome 19, which is associated with premature senescence under a short-day photoperiod in *Arabidopsis thaliana* (Suttangkakul et al. 2011), and gibberellin 3-beta-dioxygenase coding gene on chromosome 4, which is associated with plant response to light stimulus (GO:0009416), are encoded in chromosomic regions of Russian origin in the Nordic populations of Scandinavia and therefore could be also playing important roles in how Nordic trees perceive light and respond to differences in photoperiod. On the other hand, the gene model annotated as an exocyst subunit (exo70 family) protein-coding gene identified on chromosome 15 in the South of Sweden was acquired through hybridization with the Central European population. Exocyst subunits are involved in immunity to plant pathogens (Du et al. 2018) and this shared haplotype could thus confer a particular defense capacity to the hybrid population. Overall, we suggest that standing variation and BS have shaped the capacity of aspen populations to adapt to a wide range of environmental conditions during the postglacial colonization of Europe.

## Conclusion

Our large panel of resequenced aspen individuals allowed us to unravel key genomic aspects behind local adaptation across the Eurasian continent. It is clear from our results that intraspecies hybridization has played a major role homogenizing the genomic background of the species and has promoted the movement of adaptive alleles between populations. Of major relevance is the observation of a recent adaptive introgression event between Nordic populations around the *Flowering locus T* that has facilitated the survival of aspens in high latitudes. This event is, however, the only such event that can be detected in the species, showing that the emergence of advantageous alleles and their propagation is rather rare. Standing variation and its conservation through BS across lineages seems to be a more efficient way of keeping advantageous alleles and maintaining high levels of diversity. This combination of evolutionary scenarios suggests that aspens may have the capacity to adapt rapidly to new challenging environments, and this augurs well for the survival of the species under a range of possible future climate conditions.

## Materials and Methods

### Sample Collection and Sequencing

The Swedish samples used in this article belong to the SwAsp collection which has been extensively described previously (Luquez et al. 2008; Wang et al. 2018). Leaf samples were collected from the common garden at Sävar (Luquez et al. 2008) for all but two genotypes that were present only at the Ekebo common garden, for which wood tissue was sampled. Sampled tissue was stored in cool conditions until frozen at the laboratory. An additional 296 samples of *P. tremula* were obtained from across Eurasia. Trees selected from the United Kingdom were cloned from root cuttings and grown in one of two common gardens (Harrison 2009): a Scottish national aspen collection maintained by Forest Research at Bush, Midlothian, and the Eadha Enterprises clone garden at Lochwinnoch, Renfrewshire. Leaves were sampled from 125 genotypes at Bush and 15 genotypes at Lochwinnoch, representing a range of national seed zones defined by the UK Forestry Commission. Samples were freeze-dried prior to transportation to Sweden. In Norway and Russia, leaf material was sampled from 24 trees within a region. In Latvia, ten trees were sampled in each of five regions spanning the country. Fewer samples were obtained from Iceland due to the limited availability of natural stands of *P. tremula*. Due to the clonal growth of *P. tremula*, individual samples were collected with enough physical separation (0.3 km) between individuals to minimize the risk of sampling identical genets. Leaf samples were stored in silica gel before shipping to Sweden. Geographical coordinates were recorded for the sampled trees. The individuals included in the study (after the quality control steps that follow) and their geographic origins are listed (supplementary table 1, Supplementary Material online).

Total genomic DNA was extracted from frozen leaf tissue for all individuals using the DNeasy Plant Mini Prep Kit (QIAGEN, Valencia, CA). For the Icelandic samples, library preparation and sequencing were conducted by Génome Québec, Canada. Briefly, 1 µg of high-quality DNA was used for paired-end libraries construction. The 12 libraries were sequenced in three lanes of Illumina HiSeqv4 with read length of 125 bp. All other samples were subjected to paired-end sequencing using libraries with an average insert size of either 350 or 650 bp, and samples were sequenced by the National Genomics Infrastructure at Science for Life Laboratory, Stockholm, on an Illumina HiSeq 2000 or Illumina HiSeq X platform to a mean per-sample depth of approximately 17×.

### Mapping and SNP Calling

Resequenced accessions were mapped against the reference genome of *P. tremula* v2.0 (Schiffthaler et al. 2019), using BWA [v0.7.17; (Li and Durbin 2009)]—mem alignments for paired-end reads using default parameters. Postmapping filtering removed reads with MQ < 20 (using samtools v1.10; Li et al. 2009). Depth and breadth of coverage were assessed in order to confirm all samples had a minimum coverage of 8× (supplementary table 1, Supplementary Material online). Finally, before the variant calling step, we tagged duplicate reads (using Picard MarkDuplicates v2.10.3; Picard Toolkit.” 2019. Broad Institute, GitHub Repository: http://broadinstitute.github.io/picard/ (last accessed August 9, 2021) and found that they did not exceed 14% of the sequencing reads in individual libraries (ranging from 3% to 13.8%).

We used GATK v3.8 to call variants (DePristo et al. 2011). First, we performed a local realignment around indels with RealignerTargetCreator and IndelRealigner (using default parameters). We called per-sample variants using HaplotypeCaller to produce gVCF files (-ERC GVCF). Given the large number of individuals, we produced intermediate gVCF files using CombineGVCFs that were finally used in the joint-call step with GenotypeGVCFs. From the resulting VCF file, we selected SNPs using SelectVariants and filtered them with VariantFiltration (QD < 2.0; FS > 60.0; MQ < 40.0; ReadPosRankSum < −8.0; SOR > 3.0; MQRankSum < −12.5). At this point, the filtered VCF was lifted over to version 2.2 of the genome of *P. tremula*, available at ftp://plantgenie.org/Data/PopGenIE/Populus_tremula/v2.2/, using picard LiftoverVcf, which allowed for better resolution at the chromosome level. Further SNP pruning with vcf/bcftools removed positions with extreme depth values (min-meanDP 10, max-meanDP 25; these thresholds correspond to the average depth ± 1 standard deviation), absent in more than 30% of the samples, nonbiallelic or displaying an excess of heterozygosity (calculated with the plugin fill-tags of bcftools and filtered based on FDR <0.01).

### Population Structure and Admixture in *P. tremula*

We used plink (v1.90b4.9; Purcell et al. 2007) to identify linked and low-frequency SNPs (--indep-pairwise 100 10 0.2 –maf 0.05) that we removed with vcftools. We used the resulting set of pruned positions to compute the relatedness between samples from the Eurasian collection by calculating genome-wide estimates of identity by descent. We removed from our downstream analyses one sample from each pair of closely related individuals, using a threshold value of relatedness of 0.4.

Next, we used vcftools to output the genotype likelihood information contained in the pruned VCF file; the resulting beagle-formatted file was input into NGSAdmix to estimate individual admixture proportions across a varying number of ancestral populations (*K* = 3 to *K* = 6).

Finally, we assessed individual ancestries using EIGMIX implemented in SNPRelate. This eigen analysis, developed by Zheng and Weir (2016), is computationally efficient for estimating ancestral proportions by making assumptions of surrogate samples for ancestral populations. For this, we chose the Latvian, Scottish, and Russian populations as proxies of the ancestral populations (referred to as Central European, Western European, and Siberian, respectively) from which admixture was to be estimated, based on earlier estimates of the postglacial colonization history of *P. tremula* across Europe (de Carvalho et al. 2010). We used the LD/MAF-pruned SNPs and produced a GDS object for the analysis (Zheng et al. 2012).

### Gene Flow

We combined several statistics developed to identify introgressed genomic regions. These included the classic ABBA–BABA test in its developed *F*_d_ statistic form. For these calculations, we used the popgen pipeline developed by Simon Martin, available at https://github.com/simonhmartin/genomics_general (last accessed August 9, 2021). We treated each chromosome independently; the corresponding vcf file was converted to .geno format using parseVCF.py. From these files, we calculated diversity and divergence values for each subpopulation (*D*_XY_, *F*_ST_, and *P*) in nonoverlapping 10-kb windows, using the script genomics.py. Finally, we used the script ABBABABAwindows.py to compute the four-taxon *D* statistic and *f* estimators in nonoverlapping windows of 10 kb as well. For these calculations, we tested gene flow from the Russian (P3; 23 individuals) or Latvian (P3; 45 individuals) subpopulations into Northern Scandinavia (P2; 56 individuals: 34 from Northern Sweden and 22 from Norway) or Southern Scandinavian (P1; 50 from Sweden and 23 from Norway), setting the Chinese (O; 15 individuals) samples as the outgroup. Since we were interested in estimating shared variation between Russian and Northern Scandinavian individuals, we focused on the *f*_dM_ introgression statistic, described by Malinsky et al. (2015). *f*_dM_ gives positive values when introgression occurred between P3 and P2, and negative values if it occurred between P3 and P1. In order to avoid stochastic errors that could produce meaningless values, we only considered windows with at least 100 biallelic SNPs.

We also calculated the distance fraction, *d*_f_ (PopGenome; v2.7.1; Pfeifer et al. 2014; Pfeifer and Kapan 2019), which combines both *f*_d_ and distance estimations and that is less sensitive to the time of gene flow. Just as in the previous analysis, we set a four-taxon tree hypothesis to estimate the proportion of introgression from Russia into Northern Scandinavia. We calculated *d*_f_ in 10-kb windows treating each chromosome independently. We combined all 19 chromosome estimations and assigned a *Z*-score and *P*-value to each *d*_f_ value using genome-wide results and did an extra FDR correction. We selected those regions that had an FDR < 0.05.

Finally, we ran the ELAI method (Guan 2014) to confirm the introgression event on chromosome 10 and study the genomic mosaicism produced by hybridization events between the ancestral proxies at the chromosome level. We used two upper-layer clusters and ten lower-level clusters; 20 expectation–maximization steps and 100 generations of admixture between the ancestral populations that corresponded to the Russian and Latvian aspen populations for Northern and Southern Sweden, or Latvia and Scotland for Southern Norway. The plotted allele dosages correspond to the average over all the Swedish individuals from the northern population. We ran five independent EM runs. The ADD was calculated as the absolute value of the difference of allele dosage from donor population.

### Positive and Balancing Selection

We scanned the genome of *P. tremula* for signals of positive selection using a newly developed strategy called “integrated selection of alleles favored by evolution,” iSAFE (Akbari et al. 2018). This method first calculates haplotype allele frequency scores based on the presence of derived alleles in a particular haplotype, which is then used to calculate SAFE scores. These SAFE scores are in turn calculated across a region of given size in 50% overlapping windows of 300 bp to culminate in an iSAFE signal. These statistics can be calculated for large regions of phased haplotypes, which we obtained with BEAGLE v.4.1 (Browning SR and Browning BL 2007), so chromosomes were divided into 3-Mb windows for each iSAFE iteration. The iSAFE software can be set to run under a case–control mode, with the case populations being, for example, the Northern Scandinavian population, and the control population being all remaining individuals not used in the case population. We ran this screening for Northern and Southern Scandinavia, Russia, and combining all the Nordic individuals from Norway, Sweden, and Russia together. As recommended by the authors, we considered iSAFE values significant when they were >0.1.

In addition, we ran betascan (Siewert and Voight 2017), to detect possible signals of BS, by dividing the collection in six sets of hybrid populations from 1) Northern Scandinavia (Sweden and Norway), 2) Southern Sweden and 3) Southwestern Scandinavia (Southern Norway and Iceland), or donor populations from 4) Russia, 5) Latvia and 6) Scotland. We used the following betascan parameter: -fold -m 0.1, which refers to the minimum folded frequency of core SNP. We defined conserved BS sites as those SNPs with significant β-scores (FDR < 0.01) shared by all donor and hybrid populations (4,000 sites, fig. 5*A*). Using the coordinates of the gene models in the reference genome available in the gff file of the annotation, we assessed the number of genic/intergenic BS sites. To evaluate the possibility of *cis*-effects of intergenic BS sites, we binned the genome in 10-kb fragments and counted the number of BS sites in each bin. The average number of BS sites per bin (removing those with 0 sites) was 15, which we chose as the lower threshold to keep chromosome fragments considered to have BS signals. We then revised the gene content in such windows, allowing for sites to be within and up/downstream of the gene models. We used topGO v.2.36.0 (Alexa and Rahnenfuhrer 2019) to look for functional enrichments among the gene models obtained with these criteria; because of the small gene-set, no significant signals using Bonferroni corrections were detected, so we described the categories at a threshold with classic Fisher statistic of 0.05.

Finally, we combined iSAFE and β-scores with ELAI calculated ADD to assess the effect of hybridization in each scan of selection (figs. 4*C* and 5*C*). In the case of BS signals, we observed much of the significant β-scores overlapping with low ADD (<quantile 0.75), whereas only a few sites with high β-scores would be part of the top 25% ADD. The intersecting SNPs with significant β-scores and low ADD are shown in supplementary figure 11*A*, Supplementary Material online , plotted with UpSetR, (Conway et al. 2017); only 1,248 sites were shared by all hybrid and donor populations under this criteria (highlighted in green in the Manhattan plots in fig. 5*D*; supplementary fig. 11*A*, Supplementary Material online).

### Population Demographics

We chose StairwayPlot (Liu and Fu 2015) to infer changes in effective population sizes in the past. For this, the folded SFS for the tested subpopulation was calculated using ANGSD v.0.920 (Korneliussen et al. 2014) using the LD-pruned SNPs (plink --indep-pairwise 100 10 0.2). We ran independent analyses for Scandinavia, Russia and for a set of 50 randomly selected individuals from the entire collection. We generated 100 bootstrap replicates and assumed a mutation rate (μ) of 2.5e-9 and a generation time of 15 years (Ingvarsson 2008).

In addition, we aimed to confirm the directionality of the gene flow events we detected on chromosome 10 between the Russian and Scandinavian individuals. For this, we explored alternative demographic models using the diffusion approximation method of dadi (Gutenkunst et al. 2009) to analyze the SFSs of our aspen subpopulations. We used two chromosomes for this purpose, chromosome 10 and chromosome 8, since there is strong evidence of synteny between both chromosomes deriving from the 40-My-old genome duplication that are shared by all member of the genus *Populus* (Tuskan et al. 2006). We ran analyses independently for chromosomes 8 and 10 using all LD-pruned, biallelic sites including all biallelic sites present in the putatively selective sweep/introgressed region on chromosome 10. We fit 19 demographic models (supplementary fig. 11, Supplementary Material online) for the Russian and Scandinavian subpopulations using the demographic modeling workflow (dadi_pipeline) from Portik et al. (2017). We tested different classes of models: simple models of divergence with and without migration; simple models plus instantaneous size changes, ancient migration or secondary contact, ancient migration plus instantaneous size change, and island models of vicariance and founder events. In total, we tested 19 different demographic scenarios using all polymorphic sites on chromosome 10. First, we ran the general optimization routine (dadi_Run_Optimizations.py), which includes fitting the model using particular settings for a given number of replicates, then using the parameters from the best scoring replicate to seed a subsequent round of model fitting using updated settings. We used four rounds, with 10, 20, 30, and 40 replicates. The arguments controlling the steps of the optimization algorithm and the perturbation of starting parameters were maxiter=[3,5,10,15] and folds=[3,2,2,1]; we defined the extrapolation grid size to pts = [80,90,100] and projection sizes to proj = [15,15]. The optimization routine with four rounds had an important effect in minimizing differences in the likelihoods achieved at the end of the runs (supplementary fig. 12 and supplementary table 5, Supplementary Material online).

In order to generate confidence intervals for the parameters estimated from the demographic models with the highest likelihood, we created 100 bootstrap replicates of the spectra and calculated the standard deviations of the best-fit parameters using the Godambe methods (Coffman et al. 2016).

## Supplementary Material

Supplementary data are available at *Molecular Biology and Evolution* online.

## Supplementary Material

msab229_Supplementary_Data
