# Erratum to: Adaptive Introgression Facilitates Adaptation to High Latitudes in European Aspen *(Populus Tremula L.)*

**DOI:** 10.1093/molbev/msac003

**Published:** 2022-01-26

**Authors:** Martha Rendón-Anaya, Jonathan Wilson, Sæmundur Sveinsson, Aleksey Fedorkov, Joan Cottrell, Mark E S Bailey, Dainis Ruņǵis, Christian Lexer, Stefan Jansson, Kathryn M Robinson, Nathaniel R Street, Pär K Ingvarsson

*Molecular Biology and Evolution*, Volume 38, Issue 11, November 2021, Pages 5034–5050, https://doi.org/10.1093/molbev/msab229

In the originally published version of this manuscript, there was an error in the spelling of a coauthor’s name. The name should be “Dainis Ruņǵis “ instead of “Dainis Ruņis”. This error has now been corrected online.

