# Supplementary material for: Adaptive Introgression Facilitates Adaptation to High Latitudes in European Aspen (Populus tremula L.)
Source: Mol Biol Evol. 2021 Jul 30;38(11):5034–50. doi: 10.1093/molbev/msab229 (PMC8557470; doi:10.1093/molbev/msab229)
Supplement: msab229_Supplementary_Data [file msab229_supplementary_data.zip › Supplementary Material.docx]

**Adaptive introgression and standing genetic variation, two facilitators of adaptation to high latitudes in European aspen (*Populus tremula* L.)**

Martha Rendón-Anaya, Jonathan Wilson, Sæmundur Sveinsson, Aleksey Fedorkov, Joan Cottrell, Mark E.S. Bailey, Christian Lexer, Stefan Jansson, Kathryn Robinson, Nathaniel R. Street, Pär K. Ingvarsson.

**Supplementary Materials and Methods**

Batch removal on SwAsp individuals.

Given the fact that two sequencing batches from different Illumina platforms (Illumina HiSeq 2000 and HiSeq X) were used to cover the SwAsp collection, we observed noisy signals at the population structure level, presumably as a result of the different sequencing equipment and library preparation methods. By means of principal component analyses (PCA) we identified differences in the grouping of the individuals where differences among samples along PC1 and, to a lesser extent, PC2 were explained by the sequencing platform and not the geographic origin of samples (supplementary fig. 1A; PC1 separates the SwAsp collection in two sets, each corresponding to a different Illumina sequencing platform). To address these batch effects, we removed SNPs associated with both components without losing the geographic structure of the Swedish population. For this purpose, we generated a genotype file with vcftools for each chromosome. We ran independent PCA analyses on each SNP configuration (homozygous reference, heterozygous, homozygous alternative) and realized it was at the heterozygous level where the batch effect was present. Using the libraries “ggfortify”, “factoextra” and “FactoMineR”, we estimated the contribution of each variant to the components affected by the platform effect. We started by assuming a uniform contribution of each variant to each component, and removed those that would deviate from this premise in both PC1 and PC2 using the following rational:

Assuming uniformity in the contribution of the variants, we calculate the expected C value:

C=(1/nrow(SNPmatrix))*100

Threshold (T) of variants contribution both to PC1 and PC2 under uniformity:

T=((C*eig1)+(C*eig2))/(eig1+eig2)

(where eig1 and eig2 are the eigenvalues corresponding to PC1 and PC2)

For each variant, we calculate the contribution value:

var_contrib =((var_contrib[PC1]*eig1)+(var_contrib[PC2]*eig2))/(eig1+eig2)

We kept only those variants that did not contribute more than expected under the uniformity hypothesis (var_contrib <= T). While removing the batch effect using this procedure, we observed loss of more than 4e^6^ SNPs (supplementary fig. 1B).

In order to evaluate if we could remove the batch effect without compromising such a large number of variants, we assigned a p-value to the contribution of each SNP to the components using dimdesc (FactoMineR) at the chromosome level. We tested different cut-offs (supplementary fig. 2) and observed that the batch effect was removed while maintaining the geographic distribution of samples when using a p-value threshold of 0.05 for PC1 and 0.01 for PC2 (supplementary fig. 2B). With these thresholds, ~1.8 e^6^ SNPs were filtered out to remove the batch effects but without compromising the overall population structure of the samples.

**Supplementary Figures.**

**Supplementary figure 1.** SwAsp population structure before (A) and after (B) batch removal. PCAs were performed on pruned SNPs (LD=0.2; MAF=0.05); in B, SNPs contributing more than expected according to our uniformity hypothesis were removed [T=((C*eig1)+(C*eig2))/(eig1+eig2)]. In red, individuals sequenced by Illumina NextSeq; in blue, individuals sequenced by Illumina HiSeq2000.

**Supplementary figure 2.** SwAsp population structure after batch removal. PCAs were performed on pruned SNPs (LD=0.2; MAF=0.05) and applying different p-value filters to the contribution of the variants to PC1 and PC2. In red, individuals sequenced by Illumina NextSeq; in blue, individuals sequenced by Illumina HiSeq2000.


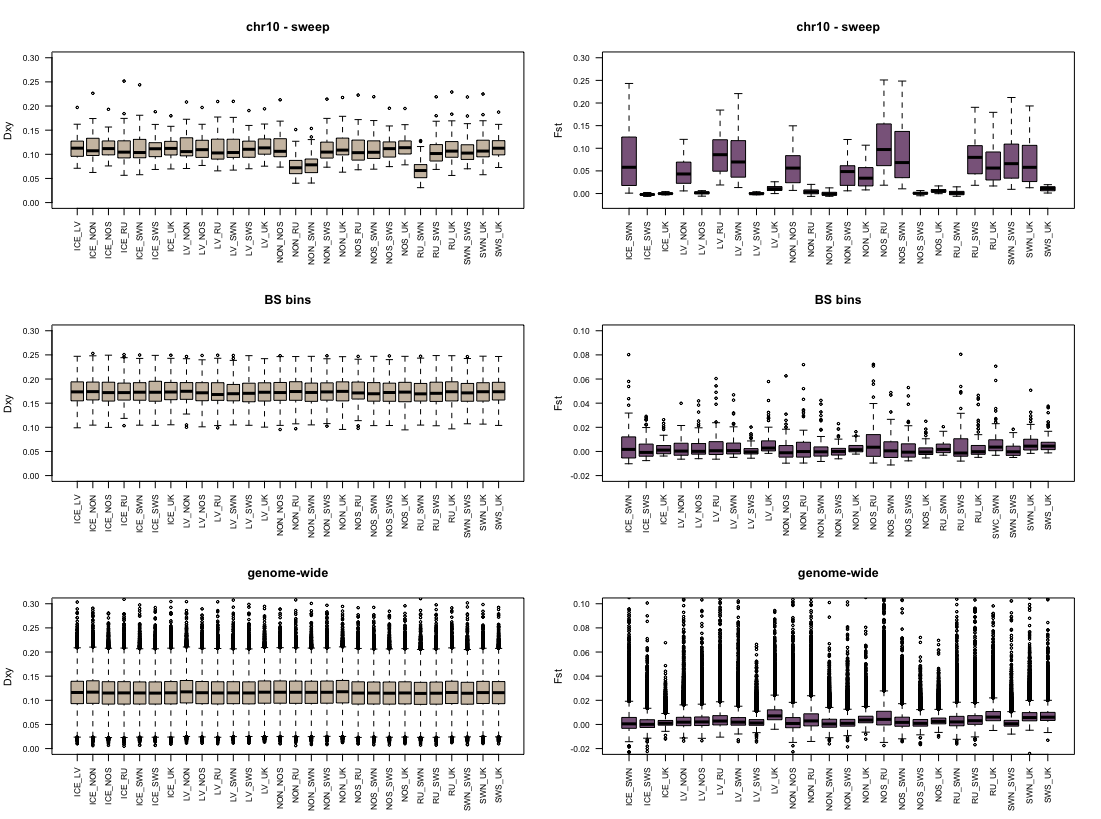


**Supplementary figure 3.** Pairwise D_XY_ and F_ST_ at genome-wide, selective sweep and BS regions.


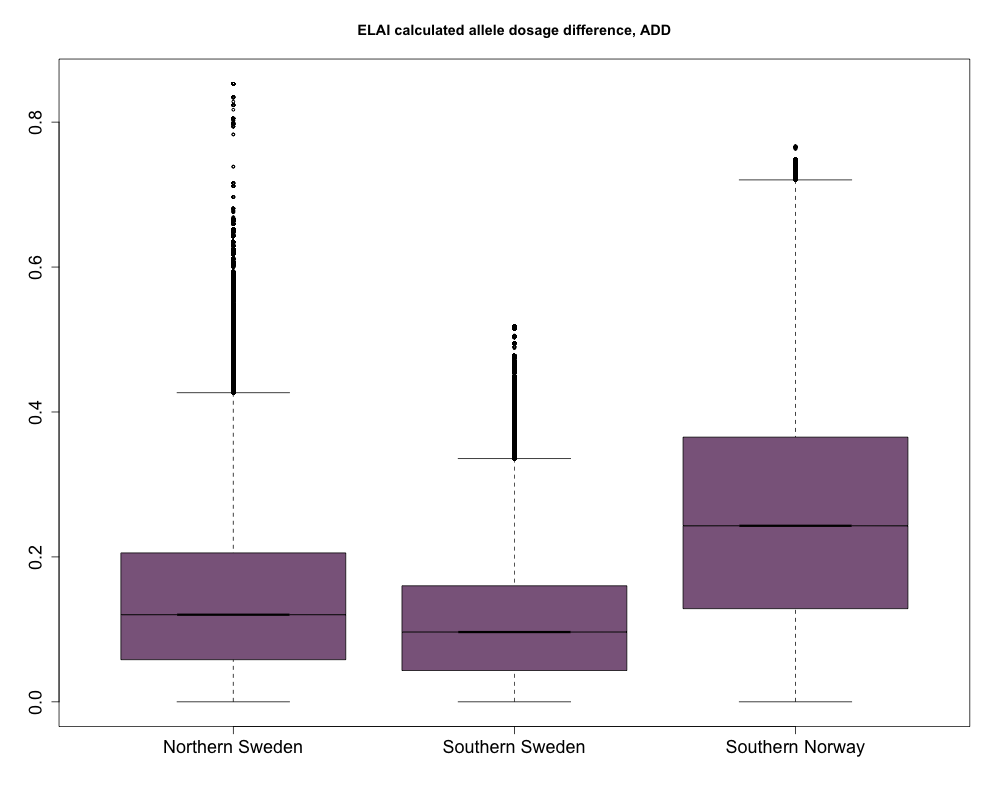


**Supplementary figure 4.** ELAI allele dosage differences in the hybrid populations.


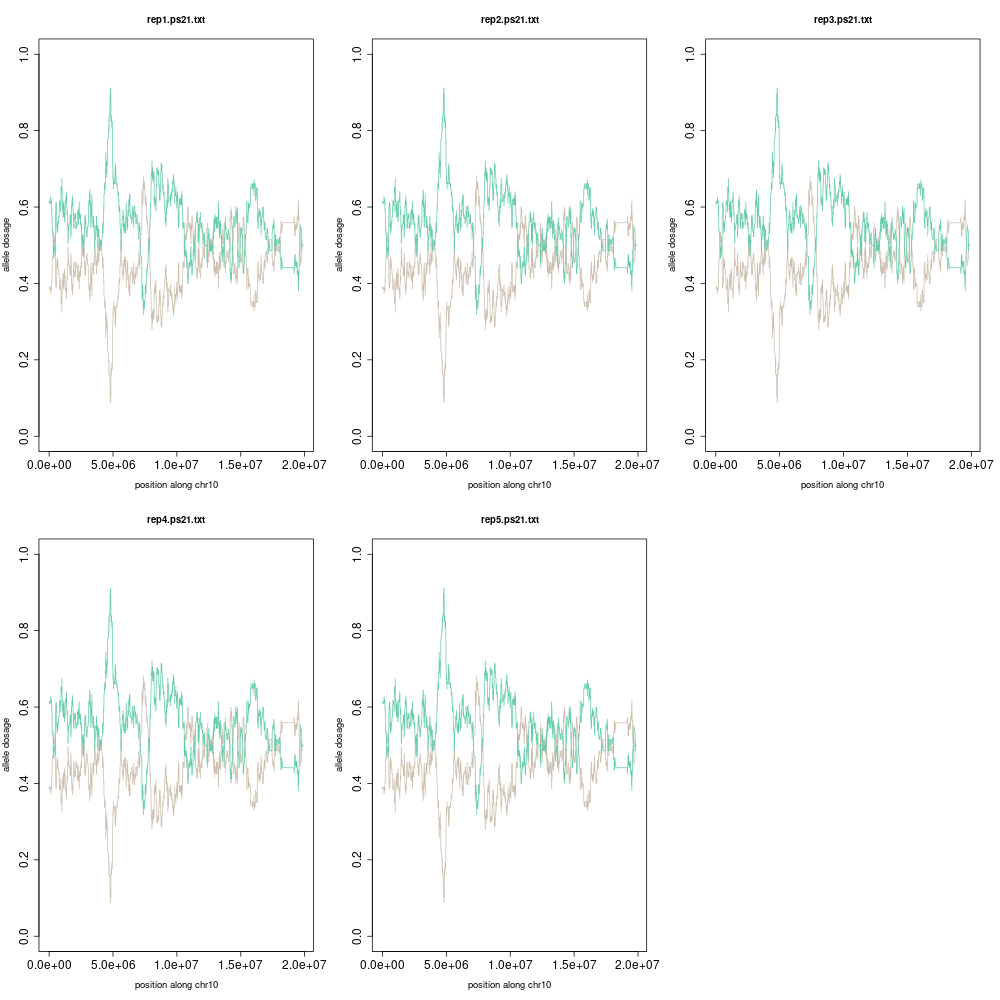


**Supplementary figure 5.** Allele dosage along chromosome 10 estimated in five independent ELAI replicates. In green, the dosage from the ancestral Russian population; in brown from the Latvian population.


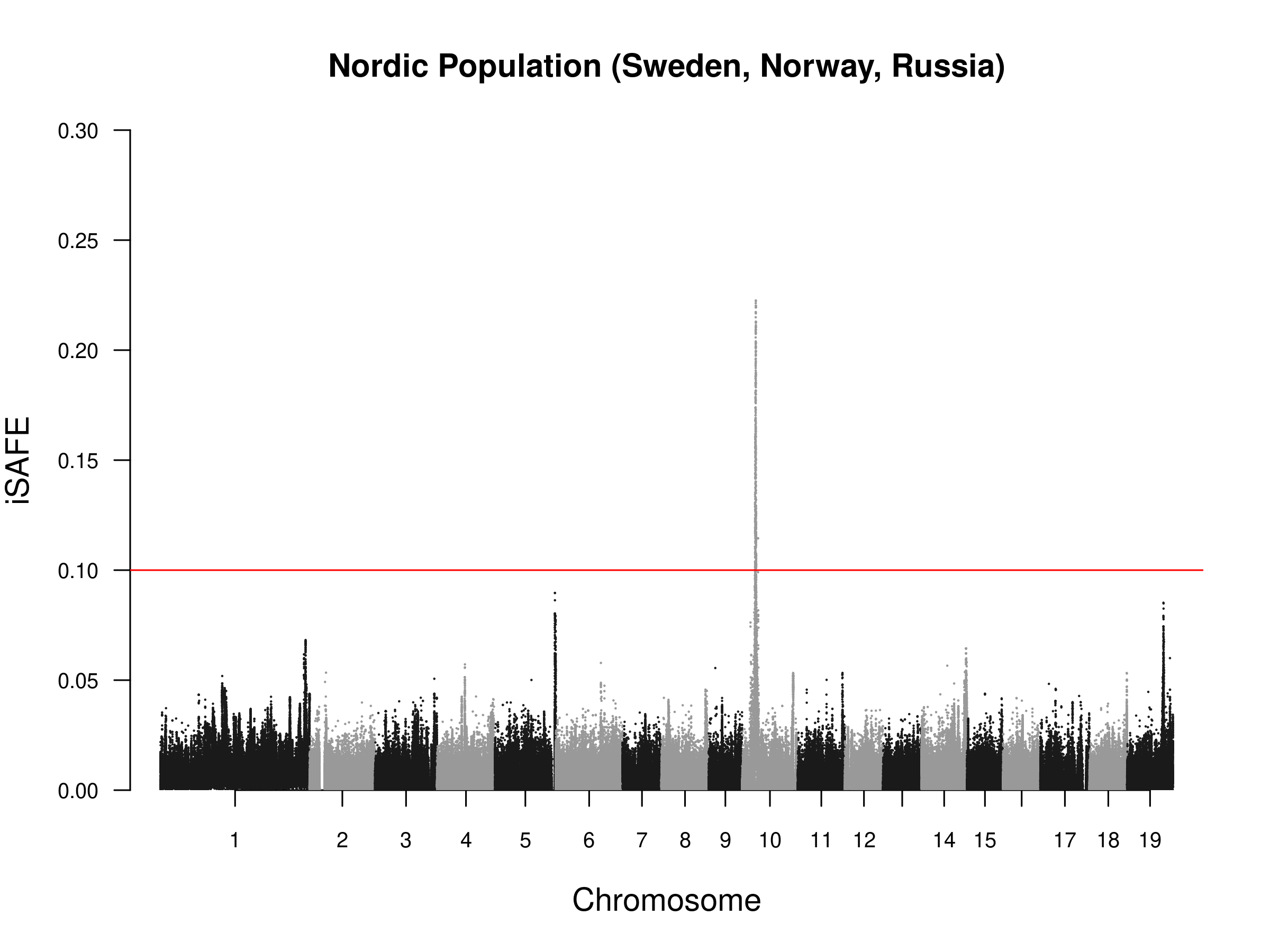


**Supplementary figure 6.** Manhattan plot of iSAFE values grouping Northern Scandinavia and Russia.


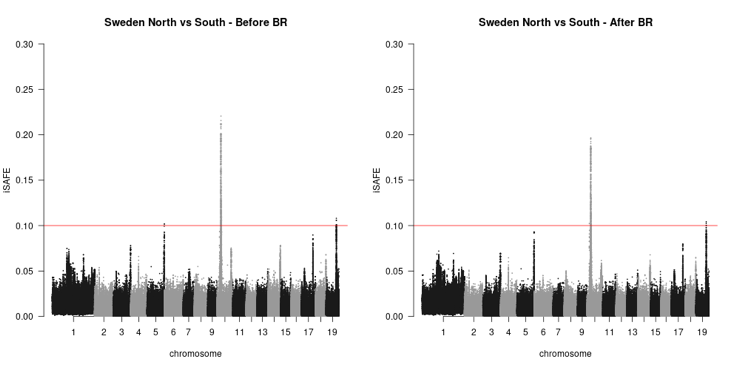


**Supplementary figure 7.** Effect of batch removal in the iSAFE analysis. Manhattan plots of iSAFE values contrasting the Northern and Southern Swedish populations in a case/control configuration, before (left panel) and after (right panel) batch removal (BR).


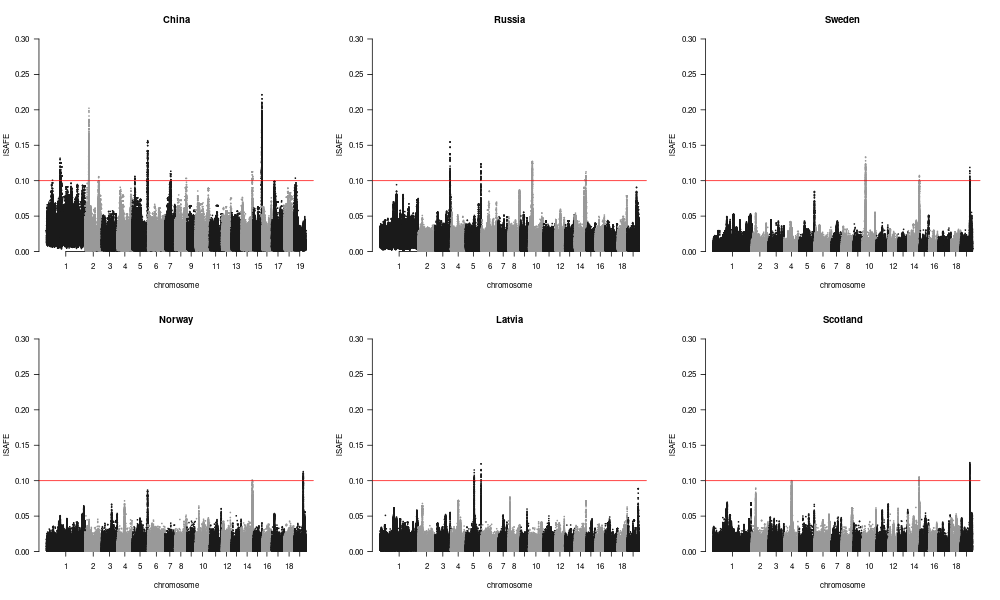


**Supplementary figure 8.** iSAFE pattern across populations.

**Supplementary figure 9.** Balancing selection signal in *P. tremula*. A. We obtained the bscores for each hybrid and ancestral population proxi using betascan. At a significance threshold of FDR<0.01 in each population and ADD<q0.75 in the hybrid populations, we calculated the intersection of SNPs. B. Comparison of b-scores between BS regions and BS regions with low ADD.

**Supplementary figure 10.** Comparison of b-scores and ADD between Northern and Southern Swedish populations. Manhattan plots show balancing selection signals in the hybrid populations. The plots are built with BS significant sites (FDR<0.01) from each population; in green we highlight the sites belonging to the 4K set of shared SNPs. Gray rectangles highlight regions with significant BS signal and deviations in ADD in one of the hybrid populations.

**Supplementary figure 11.** Demographic models tested between Northern Scandinavia and Russia.


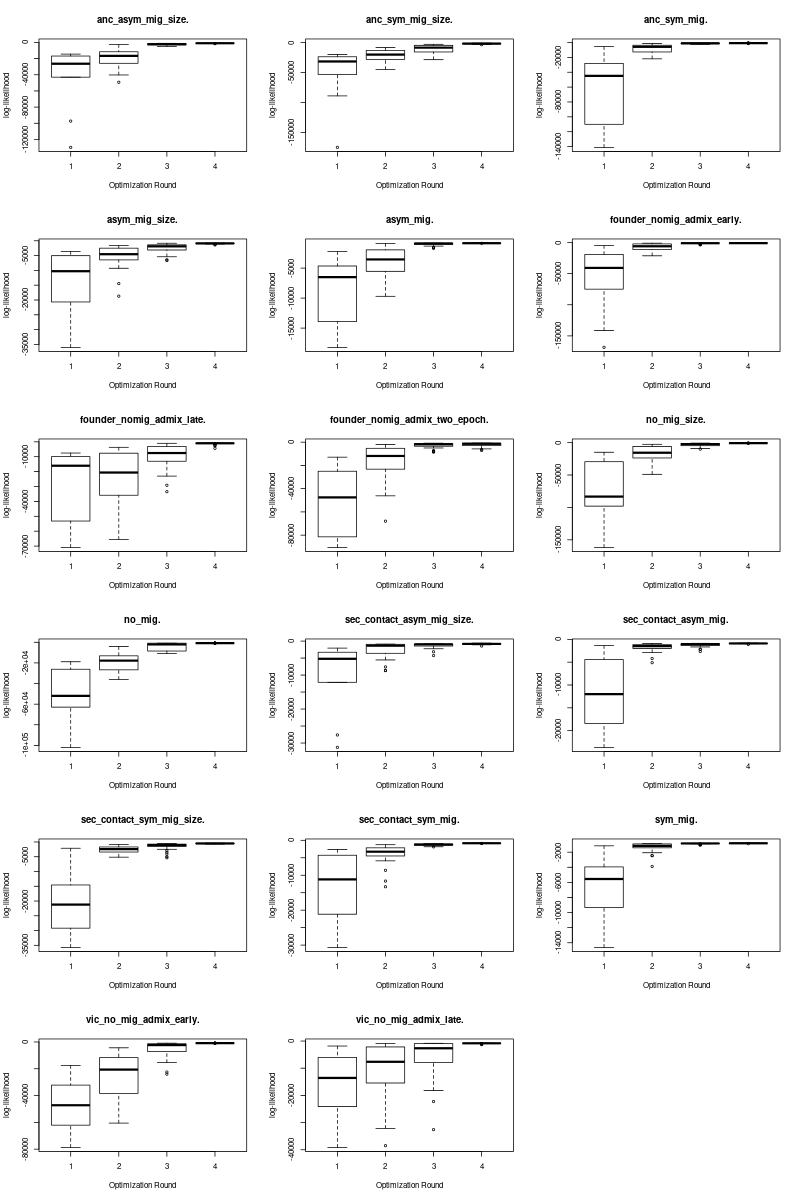


**Supplementary figure 12.** Likelihood of the tested model after each round of optimization.


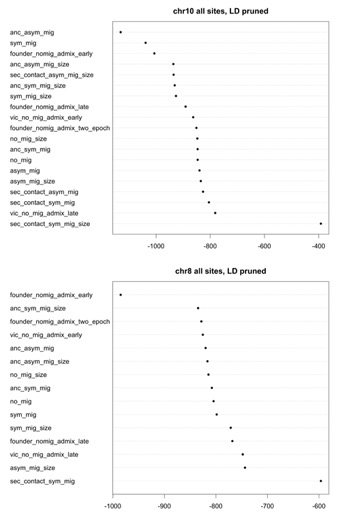


**Supplementary figure 13.** Likelihood of the demographic models. Simulations using all unlinked sites on chromosomes 8 and 10.

**Supplementary figure 14.** Likelihood of the demographic models. Simulations using sites on the selective sweep on chromosome10, changing the directionality of the migration. Ne values can be found on supplementary table 5.
